# Regulating human oocyte maturation in vitro: a hypothesis based on oocytes retrieved from small antral follicles during ovarian tissue cryopreservation

**DOI:** 10.1007/s10815-025-03483-9

**Published:** 2025-04-22

**Authors:** Jesús Cadenas, Cristina Subiran Adrados, Ajay Kumar, Bhanu Kalra, Linn Salto Mamsen, Claus Yding Andersen

**Affiliations:** 1 Laboratory of Reproductive Biology, section 5701, University Hospital of Copenhagen, Rigshospitalet, Henrik Harpestrengsvej 6A, 2100, Copenhagen, Denmark; 2 Ansh Labs LLC, 445 W. Medical Center Blvd, Webster, TX 77598 USA; 3 The Fertility Clinic, Copenhagen University Hospital Herlev, Herlev, Denmark; 4 Institute of Clinical Medicine, Faculty of Health and Medical Sciences, University of Copenhagen, Copenhagen, Denmark

**Keywords:** IVM, Human oocytes, Cumulus cells, Spent media, Hormone production, Gene expression

## Abstract

**Purpose:**

To characterize the hormonal environment in spent medium and cumulus cell gene expression during human IVM using oocytes from small antral follicles (SAFs) retrieved from surplus medulla tissue after ovarian tissue cryopreservation.

**Methods:**

Immature oocytes from surplus medulla tissue underwent 42-h IVM in media with varying FSH and LH concentrations (0, 10, 100 IU/L FSH, and 100 IU/L FSH + 100 IU/L LH). Oocyte maturation was assessed by germinal vesicle (GV), metaphase I (MI), or metaphase II (MII) stages. Gene expression of *FSHR*, *LHCGR*, *AMH*, *CYP19 A1*, and *INHA* in cumulus cells was analyzed by RT-qPCR, and GDF9, AMH, inhibin-B, inhibin-A, and total inhibin were measured in the spent media by ELISA.

**Results:**

Increased FSH concentrations downregulated *FSHR* expression and upregulated *LHCGR*, which correlated with MII transition. GDF9 concentrations in the spent medium significantly decreased with higher FSH, as did GDF9, AMH, and inhibin-B in MII oocytes. Inhibin-A levels tended to be higher in the media of MII oocytes. *FSHR* expression was positively associated with inhibin-B and negatively with inhibin-A, while *LHCGR* showed the opposite pattern and was also negatively linked to GDF9 concentration.

**Conclusion:**

FSH-induced *LHCGR* expression, along with *FSHR* downregulation, is closely linked to oocyte maturation. Reduced GDF9 secretion from oocytes facilitates *LHCGR* expression on cumulus cells, while FSH and LH collectively induced hormones like inhibin-A, which likely support oocyte maturation.

**Supplementary Information:**

The online version contains supplementary material available at 10.1007/s10815-025-03483-9.

## Introduction

In most mammalian species, assisted reproduction often involves collecting immature oocytes from ovaries obtained at an abattoir or surgically removed from animals [1, 2]. Efficient standard methods now exist to mature these oocytes in vitro (IVM), consistently resulting in successful IVF and healthy offspring [3]. However, in humans, the development of effective IVM methods has been hampered by scarcity of immature oocytes for research, essential to delineate the species-specific processes regulating human oocyte maturation [4]. The introduction of fertility preservation through ovarian tissue freezing has changed this scenario [5], providing a relatively high number of immature human oocytes from the medulla tissue, which is normally discarded during the procurement of the cortex for freezing [6, 7].

Oocytes collected from the medulla of human ovaries originate in follicles that are considerably smaller than those normally used in human IVM studies. Formerly, IVM methods utilized oocytes from follicles with a diameter of around 8–14 mm, often following a two-to-three-day stimulation with exogenous gonadotropins and/or a bolus trigger of hCG (e.g., 6.500 IU hCG) [8, 9] usually omitting oocytes from follicles with a diameter of less than 6 mm due to their limited developmental potential [10, 11]. In contrast, oocytes from follicles collected from the medulla during fertility preservation are typically below 6 mm in diameter. Although these oocytes were traditionally disregarded, they have shown potential for fertility and meet several quality markers ascribed to oocyte viability: (i) The diameter of these immature oocytes is similar (around 115 µm) to those from traditional IVF treatments [12]. The oocyte diameter has been associated with its ability to sustain transformation from the germinal vesicle (GV) stage to the metaphase of the second meiotic division (MII stage) and for further embryonic development [13]; (ii) The atresia rate of follicles used in standard ovarian stimulation protocols is around 55–77%, whereas the atresia rate for small antral follicles (SAFs) with a diameter of just a few millimeters is only 15–24% [14], suggesting higher viability of these oocytes from smaller follicles, although they generally exhibit less competence; (iii) The density of FSH receptor (FSHR) expression on granulosa cells likely reflects their sensitivity to FSH stimulation. Studies indicate that *FSHR* expression decreases significantly as human follicles grow from a few millimeters to over 6 mm [15, 16]. It is therefore likely that cumulus-oocyte-complexes (COCs) from SAFs respond better to FSH stimulation than COCs from larger follicles.

Our research focuses on developing an efficient human IVM platform using oocytes from SAFs. We have previously determined that the FSH concentration is crucial for MII transition, with concentrations exceeding 75 IU/L yielding better results in the presence of LH [17]. Combining FSH with LH (100 IU/L) appears to enhance efficacy, and there is a strong inverse relationship between gene expression of *FSHR* and LH receptor (*LHCGR*) during the IVM period, which is associated with inducing oocyte maturation [17]. Additionally, we observed that the oocyte-secreted TGF-β members, GDF9 and BMP15, exhibit distinct patterns during IVM, as measured by secretion to the spent medium, with lower concentrations in medium from oocytes that successfully transition to MII compared to those that do not when both FSH and LH were added to the IVM medium [18].

In the present study, we aim to further characterize the hormonal environment in the spent medium and the gene expression profiles of cumulus cells during human IVM using oocytes from SAFs collected during fertility preservation and ovarian cortical preparation.

## Materials and methods

### Study approval

The project (JH- 2–2011–044) was approved by the Scientific Ethical Committee for the Capital Region of Denmark and conducted according to the guidelines of the Declaration of Helsinki. All patients gave informed consent to donate their surplus ovarian tissue for research purposes.

### Patients

A total of 20 patients (mean age 29 years; range 16–37) who underwent unilateral ovariectomy and ovarian tissue cryopreservation (OTC) for fertility preservation were included in the study. The indications for fertility preservation were breast cancer (*n* = 13), brain cancer (*n* = 1), stomach cancer (*n* = 1), lymphoma (*n* = 1), and sclerosis (*n* = 1). Spent medium from an additional three patients (23–34 years, breast cancer (*n* = 2), lymphoma (*n* = 1)) was preliminarily measured for the concentration of inhibin-B (*n* = 29 samples), and data were also included. The phase of the patient’s menstrual cycle and their fertility history were not recorded.

### Ovary transport and oocyte collection

After ovariectomy, ovaries were transported in Custodiol® HTK (Dr Franz Köhler Chemie GmbH., Bensheim, Germany), either at 37 °C from the local hospital (a 10 min transit) or on crushed ice from collaborating hospitals (2–5 h transport) [19]. After the isolation of the ovarian cortex for OTC, all dishes containing the surplus medulla tissue in DMEM/F- 12 medium (GIBCOTM, Life Technologies, Paisley, UK) were carefully examined for the presence of immature oocytes under a stereomicroscope (Leica MZ12, Germany) within a flow hood featuring a heated tabletop at 37 °C. Recovered oocytes were placed in holding medium consisting of McCoy’s 5*α* plus 25 mM HEPES (Invitrogen, GIBCO™) with 10 µg/mL insulin, 5.5 µg/mL transferrin, 6.7 ng/mL selenium (ITS; Invitrogen Co., GIBCO™), 2 mM Glutamax (GIBCO™), 5 mg/mL human serum albumin (HSA; CSL Behring 20%, Germany), and 0.05 mg/mL penicillin/streptomycin (GIBCO™). Only oocytes with clear signs of degeneration, such as shrunken and darkened cytoplasm, were excluded from the study.

### *Oocyte *in vitro* maturation and collection of cumulus cells and spent media*

Oocyte IVM and collection of cumulus cells and spent media were performed as described previously with minor modifications [18]. Briefly, oocytes were divided into three categories according to the size of the cumulus cell mass: large cumulus cell mass (L-COCs), small cumulus cell mass (S-COCs), and naked oocytes (NO) [17]. The oocytes were washed twice in IVM medium with no gonadotropins, which consisted of MediCult IVM system (Origio A/S, Denmark) supplemented with 10 mg/mL HSA and 1 µg/mL human recombinant Midkine (SRP3114, Sigma-Aldrich, USA) and then individually transferred to fresh 25-µL drops of IVM medium with increasing concentrations of human rFSH (Rekovelle, Ferring, Copenhagen, Denmark) alone or in combination with 100 IU/L human recombinant luteinizing hormone (rLH) (Luveris, Serono, Germany) as follows: IVM medium with no gonadotropins (No GT), IVM medium with 10 IU/L rFSH (FSH10), IVM medium with 100 IU/L rFSH (FSH100), or IVM medium with both 100 IU/L rFSH and 100 IU/L rLH (FSH100 + LH100). For each patient, L-COCs, S-COCs, and NO were equally distributed among the different treatments. All oocytes were incubated under paraffin oil (Origio A/S, Denmark) for 42 h at 37 °C with 5% CO_2_ in air. After that, COCs were denuded with a 130–133 µm denudation pipette (Vitrolife, Gothenburg, Sweden) and classified based on their maturation stage using an inverted microscope (Carl Zeiss Axiovert 135, Germany; × 20 magnification). Maturation stages included GV, metaphase I (MI), MII, with the presence of a first polar body, or degenerated (DEG), which were discarded. The spent media and/or cumulus cells from non-degenerated GV, MI, and MII oocytes after IVM were individually collected and stored at − 80 °C for further analyses.

### Multiplex quantitative real-time PCR (RT-qPCR) in cumulus cells

Total RNA was individually extracted and purified from cumulus cells with TRIzol® reagent (Ambion, Life Technologies, USA) and 1-bromo- 3-chloropropane (Sigma-Aldrich, USA) and subsequently, with RNeasy® Minikit 250 (Qiagen, Denmark) according to the manufacturer’s instructions. All steps were performed on ice. Due to the limited amount of RNA, only samples from L-COCs were included. The quality and quantity of the isolated RNA were evaluated using the Agilent RNA 6000 Pico kit and the Agilent Bioanalyzer 2100 (Agilent Technologies, Santa Clara, CA, USA). Only samples with an RNA integrity value (RIN) ≥ 5 (mean RIN 8.7) were selected (*n* = 41; GV, *n* = 9; MI, *n* = 3; MII, *n* = 29). For each selected sample, first-strand cDNA was prepared using the High Capacity cDNA Reverse Transcription Kit (Applied Biosystems, Foster City, CA, USA) following the manufacturer’s instructions. The multiplex RT-qPCR analysis was done by TaqMan® technology using the TaqMan™ Fast Advanced Master Mix (Applied Biosystems, Foster City, CA, USA). The following TaqMan probes were used: LH receptor (*LHCGR*; #Hs00174885_m1), FSH receptor (*FSHR*; #Hs01019695_m1), anti-Mullerian hormone (*AMH*; #Hs00174915_m1), aromatase cytochrome p450 family 19 subfamily a polypeptide 1 (*CYP19 A1*, #Hs00903411_m1), inhibin subunit alfa (*INHA*, #Hs00171410_m1), and glyceraldehyde 3-phosphate dehydrogenase (*GAPDH*; #Hs02786624_g1) and as the reference gene [20]. All samples were run in duplicates and normalized to *GAPDH*. The relative expression levels were quantified according to the comparative cycle threshold method (QuantStudioTM 3 Real-Time PCR system, Applied Biosystems by Thermo Fisher Scientifics).

### Determination of GDF9, AMH, inhibin-A, inhibin-B, and total inhibin by ELISA

Concentrations of GDF9, AMH, inhibin-A, inhibin-B, and total inhibin were measured using ELISA assays in single drops of spent media, L-COCs (*n* = 68), S-COCs (*n* = 40), and NO (*n* = 19). The measurements were performed using ELISAs from Ansh Labs, Webster, Texas, USA, following the manufacturer’s instructions. The characteristics of the ELISAs used are listed in Supplementary Table 1.

### Statistical analysis

For oocyte IVM and gene expression in cumulus cells, the statistical analysis was performed using R version 4.2.2 [21] and the packages “lme4” [22] and “GLMMadaptive” [23]. In vitro maturation was analyzed by fitting a logistic mixed model with maturation as a binary outcome, patient as a random effect, and treatment as a fixed effect. Separate models were fitted to compare maturation rates on L-COCs, S-COCs, and NO. To evaluate the differences between L-COCs, S-COCs, and NO, an independent logistic mixed model was fitted with maturation as a binary outcome, patient as a random effect, and size of cumulus as a fixed effect. Gene expression of cumulus cells from L-COCs after IVM was modelled by a linear mixed model with gene expression as a continuous outcome, patient as a random effect, and treatment as a fixed effect. Spearman correlations between hormones and growth factors measured in the spent medium and gene expression in the cumulus cells, as well as ANOVA and *t* tests for comparison of concentrations, were performed using GraphPad Prism 8. For Spearman correlations analysis, ELISA values below the detection limit were ascribed a value of half the detection limit, and values without valid output were omitted. All *P*-values below 0.05 were regarded as significant.

## Results

### *Oocyte recovery and *in vitro* maturation*

A total number of 271 (from 17 patients included in the IVM testing; mean recovery rate: 16 oocytes/patient, range 3–29) + 29 (additional only inhibin-B preliminary measured from three patients not included in the IVM testing, i.e., only GV and MII from L-COCs with 100 IU/L of both FSH and LH during IVM) immature oocytes were collected ex vivo from SAFs present in surplus medulla tissue during OTC: L-COCs (*n* = 123), small-COCs (*n* = 81), and NO (*n* = 67). The oocyte maturation rates within each treatment and oocyte category are shown in Table 1.. Overall, 47% of the oocytes reached the MII stage after IVM, and the maturation rates significantly increased with increasing the size of cumulus mass (24%, 46%, and 61% in NO, S-COCs, and L-COCs, respectively) (NO vs. S-COCs, *P* = 0.004; NO vs. L-COCs, *P* = < 0.001; and S-COCs vs. L-COCs, *P* = 0.03). Moreover, the addition of 100 IU/L of rFSH alone significantly augmented the maturation rates of L-COCs when compared to the treatment with no GT (from 49 to 76% MII-rate; *P* = 0.02), while 10 IU/L rFSH or the combination of 100 IU/L rLH and 100 IU/L rFSH did not reach significance.

**Table 1.** Oocyte nuclear maturation rates according to IVM treatment and type of COC

IVM treatment	Metaphase II rate (%)			
	NO	S-COCs	L-COCs	Overall
No GT	18 (3/17)^a^	47 (9/19)	48 (15/31)*^b^	40 (27/67)
FSH10	41 (7/17)	48 (10/21)	52 (16/31)	49 (33/69)
FSH100	19 (3/16)^a^	43 (9/21)^a^	76 (22/29)*^b^	52 (34/66)
FSH100 + LH100	18 (3/17)^a^	45 (9/20)	69 (22/32)^b^	49 (34/69)
**Overall**	24 (16/67)^a^	46 (37/81)^b^	61 (75/123)^c^	47 (128/271)

No GT: group with no gonadotropins; FSH10: group with 10 IU/L rFSH; FSH100: group with 100 IU/L rFSH; FSH100 + LH100: group with both 100 IU/L rFHS and 100 IU/L rLH; NO: naked oocytes, S-COCs: small cumulus-oocyte complexes (COCs); L-COCs: large COCs

^*^Significantly different within a column (*P* = 0.02)

^a,b,c^Different lowercase letters indicates significant differences within a row: No FSH/LH (*P* = 0.03); FSH100, L-COCs vs. naked (*P* = 0.001) and S-COCs (*P* = 0.02); FSH100 + LH100 (*P* = 0.002); overall, naked vs. S-COCs (*P* = 0.004) and L-COCs (*P* = < 0.001), and S-COCs vs. L-COCs (*P* = 0.03)

### Gene expression data

**Fig. 1:**
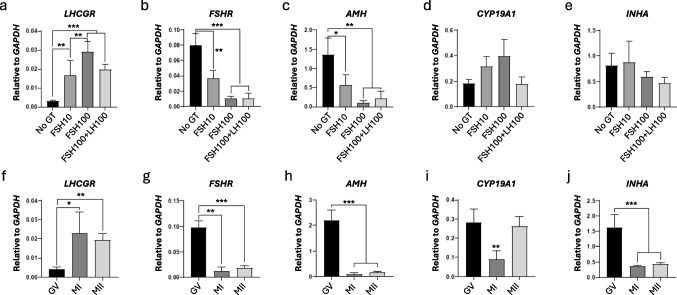
Relative expression of selected genes in cumulus cells from COCs after IVM in the presence of different gonadotropin supplementation (upper figures **a**–**e**) and according to maturation stage (GV, MI, and MII; lower figures **f**–**j**). No GT: no gonadotropins; FSH10: 10 IU/L rFSH; FSH100: 100 IU/L rFSH; FSH100 + LH100: both 100 IU/L rFSH and 100 IU/L rLH. *LHCGR*, luteinizing hormone receptor; *FSHR*, follicle-stimulating hormone receptor; *AMH*, anti-Müllerian hormone; *CYP19 A1*, aromatase cytochrome p450 family 19 subfamily a polypeptide 1; *INHA*, inhibin subunit alpha. *P < 0.05, **P < 0.001, ***P < 0.0001

The relative gene expression of *LHCGR, FSHR, AMH, CYP19 A1*, and *INHA* was measured in cumulus cells after the IVM period (*n* = 40), all L-COCs (Fig. 1). The results are categorized based on IVM treatment (Fig. 1a–e) and meiotic progression from GV to MII (Fig. 1f–j).

The expression *LHCGR* was significantly upregulated with the addition of rFSH to the IVM medium. This upregulation was more pronounced as the FSH concentration increased from 10 IU/L (*P* = 0.003) to 100 IU/L (*P* < 0.001) (Fig. 1a). In contrast, the opposite was observed for *FSHR* and *AMH* (Fig. 1b, c), i.e., a downregulation of the gene expression when increasing rFSH concentration from 10 IU/L (*P* = 0.001 for *FSHR* and *P* = 0.04 for *AMH*) to 100 IU/L (*P* < 0.001 for *FSHR* and *P* = 0.002 for *AMH*), whereas no significant differences were seen for the gene expression of *CYP19 A1* and *INHA*. The combination of 100 IU/L rLH and 100 IU/L rFSH showed no further changes in gene expression compared to the treatment with 100 IU/L FSH alone. Moreover, the expression of *CYP19 A1* (Fig. 1d) and *INHA* (Fig. 1e) was not affected by the IVM treatment (*P* > 0.05).

During the progression of meiosis from the GV to the MI and MII stages, the gene expression pattern of *LHCGR* was significantly upregulated in cumulus cells that sustained meiotic resumption (Fig. 1f). In contrast, *FSHR*, *AMH,* and *INHA* were significantly downregulated in cumulus from complexes in which the oocyte resumed meiosis (Fig. 1g, h, j). The differences in gene expression were more pronounced between the GV and the MII stages as compared to the GV and MI stages: GV vs. MI (*P* = 0.04 for *LHCGR*, *P* < 0.001 for *FSHR*, and *P* < 0.001 for *AMH*); GV vs. MII (*P* < 0.001 for *LHCGR*, *P* < 0.001 for *FSHR*, and *P* < 0.001 for *AMH*).

Furthermore, a significant downregulation of *INHA* was observed in cumulus cells during oocyte maturation: MI (*P* < 0.001) and MII (*P* < 0.001) versus GV (Fig. 1j). Also, *CYP19 A1* was significantly downregulated in cumulus cells from MI oocytes (*P* = 0.005) (Fig. 1i), although no significant differences were observed between GV and MII (*P* > 0.05), indicating that this downregulation was not directly related to oocyte maturation.

### Concentrations of growth factors and hormones in IVM spent media in relation to oocyte maturation and media composition

The concentrations of GDF9, AMH, inhibin-B, inhibin-A, and total inhibin were measured in 127 IVM spent media plus an additional 29 spent media in which only inhibin-B were measured. Table 2 shows the overall data, and supplementary Tables 2–5 provide more detailed results for each factor.

**Table 2:** Concentrations (ng/mL) of GDF9, AMH, inhibin-B, inhibin-A, and total inhibin in spent IVM drops after IVM according to oocyte maturation and IVM media composition (mean ± SEM)

	Maturation stage			IVM media composition (IU/L)			
	GV	MI	MII	0 FSH	10 FSH	100 FSH	100 FSH + 100 LH
**GDF 9**	3.3 ± 0.7^**A**^ (*n* = 39)	5.3 ± 1.2^**A**^ (*n* = 16)	1.4 ± 0.2^**B**^ (*n* = 72)	4.2 ± 0.9^**a**^ (*n* = 29)	2.3 ± 0.6^**ab**^ (*N* = 39)	1.7 ± 0.3^**b**^ (*N* = 31)	1.8 ± 0.3^**b**^ (*n* = 28)
**AMH**	279 ± 100^**A**^ (*n* = 31)	118 ± 48^**AB**^ (*n* = 13)	72 ± 11^**B**^ (*n* = 62)	103 ± 26 (*n* = 24)	91 ± 24 (*N* = 28)	108 ± 53 (*N* = 28)	252 ± 108 (*n* = 26)
**Inhibin-B**	46 ± 9.3^**A**^ (*n* = 28)	23 ± 5.5 (*n* = 13)	11 ± 0.9^**B**^ (*n* = 64)	26 ± 5.0 (*n* = 17)	20 ± 4.7 (*n* = 26)	12 ± 3.1 (*N* = 23)	20 ± 3.8 (*n* = 39)
**Inhibin-A**	3.8 ± 1.7 (*n* = 10)	ND	7.6 ± 1^**α**^ (*n* = 33)	2.5 ± 0.8 (*n* = 3)	5.8 ± 1.3 (*n* = 7)	6.6 ± 1.3 (*n* = 12)	9.9 ± 1.5^**β**^ (*n* = 21)
**Total-Inhibin**	4.7 ± 1.3^**A**^ (*n* = 25)	4.3 ± 1.4 (*n* = 8)	2.5 ± 0.3^**B**^ (*n* = 51)	2.2 ± 0.4 (*n* = 15)	2.4 ± 0.5 (*n* = 23)	2.7 ± 0.8 (*n* = 23)	5.4 ± 1.2 (*n* = 23)

Different uppercase letters (A, B) indicate significant differences between oocyte maturation stages (GV, MI, and MII) (*P* < 0.05). Different lowercase letters (a, b) indicate differences among different IVM media composition (*P* < 0.05)

*ND* no data

^**α**^*P* = 0.07

^**β**^*P* = 0.09

### GDF9

The concentrations of GDF9 were measured in all 127 spent media from IVM (Table 2). The results highlight the relationship between GDF9 concentrations, oocyte maturational status, and media composition. Overall, a significant reduction in GDF9 was observed in spent media from oocytes that reached the MII stage compared to those that remained in the GV stage. Notably, GDF9 concentrations were significantly lower in spent medium from NO, peaking in S-COCs, and reaching an intermediate level in L-COCs, with all groups showing significant differences (Supplementary Table 2). Furthermore, increasing concentrations of FSH in the IVM medium significantly downregulated GDF9 secretion. In contrast, the addition of LH did not affect GDF9 secretion to the spent medium (Table 2). Interestingly, the GDF9 concentration in the spent medium from oocytes that reached the MII stage did not vary with different FSH concentrations, which contrasts the results observed in the GV and MI groups (Supplementary Table 2). However, these latter groups contained relatively few observations.

### AMH

The concentrations of AMH were measured in 106 samples of spent media from IVM, with 21 samples showing concentrations below the assay’s detection limit. Overall, a significant reduction in AMH levels was observed in the spent media from oocytes that reached the MII stage compared to those that remained in the GV stage (Table 2). Cumulus cell–secreted AMH significantly increased from NO to S-COCs and L-COCs, with peak levels observed in the spent medium from L-COCs that remained in the GV stage during IVM (Supplementary Table 3). In general, the FSH concentrations in the IVM medium did not impact AMH secretion in cumulus cells when the corresponding oocyte reached the MII stage (Table 2).

### Inhibin-B

The concentrations of inhibin-B were measured in 105 samples from IVM spent media, whereas 51 samples showed a concentration below the detection limit of the assay. Overall, a significant increase in inhibin-B levels was observed in spent media from oocyte complexes that remained in the GV stage, compared to those at the MI and MII stages (Table 2), like that of AMH. However, the NO did not secrete measurable concentrations of inhibin-B (Supplementary Table 4). Furthermore, the secretion of inhibin-B increased significantly with the number of surrounding cumulus cells from S-COCs to L-COCs, peaking in spent medium from L-COCs that remained in the GV stage during IVM (Supplementary Table 4). The inhibin-B concentration was similar irrespective of FSH concentration in the spent medium and oocyte maturational status (Table 2).

### Inhibin-A and total inhibin

Valid measurements for inhibin-A and total inhibin in spent medium were obtained for only a fraction of the samples (Supplementary Table 5), with overall results showing valid measurements presented in Table 2. Total inhibin levels showed a pattern like that of GDF9, AMH, and inhibin-B, with reduced levels in media in which the oocyte progressed to the MII stage. Interestingly, inhibin-A displayed an opposite trend, with increased concentrations in media containing FSH and LH (*P* = 0.09) and an oocyte that sustained meiosis (*P* = 0.07) (Table 2).

### Associations between gene expressions and ELISA measurements in IVM spent medium

Spearman correlation coefficients (*r*) and *P*-values between different TGF-β members and hormones expressed in cumulus cells and in spent medium from IVM are shown in Table 3.

**Table 3:**
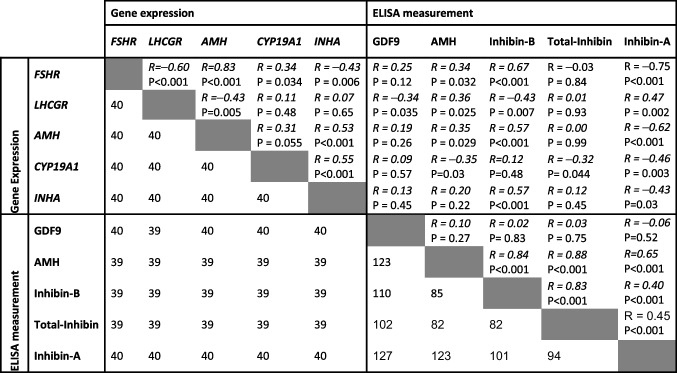
Spearman correlation analysis of gene expression in cumulus cells and ELISA determined hormone concentrations in spent medium after IVM

The corresponding cell in the lower part of the table shows the number of observations used for the analysis. In the analysis of LHCGR and GDF9 associations, one outlier was omitted. FSHR: FSH receptor; LHCGR: LH receptor; AMH: anti-Mullerian hormone; CYP19 A1: aromatase cytochrome p450 family 19 subfamily a polypeptide 1; INHA: inhibin subunit alfa

The gene expression of the *FSHR* in cumulus cells showed a significant negative association with *LHCGR* and *INHA* and significant positive associations with *AMH* and *CYP19 A1.* Notably, a strong positive association was observed between *FSHR* and inhibin-B concentrations in the spent medium, while inhibin-A showed a similarly strong negative association.

In contrast, *LHCGR* expression exhibited significant negative associations with *AMH* gene expression, and GDF9 and inhibin-B in the spent medium, but significant positive association with inhibin-A and AMH measured in the spent medium.

The gene expression of *AMH* was significantly positively associated with *INHA,* as well as with inhibin-B and AMH in the spent medium. Additionally, a significant negative association was found between *AMH* and inhibin-A protein in spent medium.

The expression of *CYP19 A1* showed a significant positive correlation with *INHA*, but significant negative associations with inhibin-A, AMH, and total inhibin protein in spent medium.

Gene expression of *INHA* was significantly positively associated with inhibin-B concentrations and significantly negatively associated with levels of inhibin-A in the spent medium.

Protein concentrations of AMH were significantly positively associated with inhibin-B, inhibin-A, and total inhibin protein concentrations in spent medium. Inhibin-B concentrations were significantly associated with both inhibin-A and total inhibin. Total inhibin was significantly associated with inhibin-A concentrations in the spent medium, while GDF9 was not significantly associated with any other hormones in the spent medium.

**Fig. 2:**
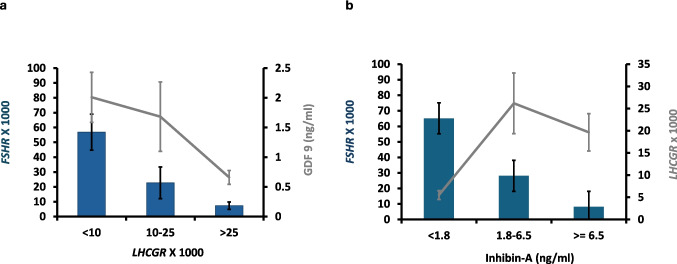
**a** *LHCGR* gene expression in relation to *FSHR* gene expression (Spearman association *R* = − *0.60 P* < 0.001) and the concentration of GDF9 in spent medium after IVM (Spearman association *R* = − 0.35, *P* < 0.035). **b** Concentration of inhibin-A in spent medium after IVM in relation to *FSHR* and *LHCGR* gene expression (Spearman associations: *FSHR*: *R* = − 0.75, *P* < 0.001; *LHCGR*: *R* = 0.42, *P* < 0.002)

The significant negative association between gene expression of *FSHR* and *LHCGR* is illustrated in Fig. 2a, which also highlights the significant negative association between the GDF9 concentration and *LHCGR* gene expression. In Fig. 2b, the significant positive association between *FSHR* expression and inhibin-A protein concentration in the spent medium is depicted, including the positive association between *LHCGR* expression and the concentration of inhibin-A in the spent medium.

## Discussion

This study demonstrates that regulation of human oocyte maturation differs significantly between in vitro and in vivo conditions. Notably, the levels of FSH in the IVM medium exceed those observed during the normal midcycle surge of gonadotropins by almost an order of magnitude, which during the IVM procedure stimulate cumulus cells to alter the hormonal secretions and composition of the spent medium and furthermore advance MII transition. In vivo, mural granulosa cells and cumulus cells undertake distinct roles and cooperate to advance meiosis. However, during IVM, only cumulus cells are present. Despite this, high levels of FSH (i.e., > 75 IU/L) can augment oocyte maturation in the absence of mural granulosa cells. The present study attempts to delineate the mechanisms operating during human IVM with COCs from SAFs, demonstrating that adequate FSH stimulation creates a hormonal environment within the small 25 µl IVM droplet that is highly variable but also associated with successful oocyte maturation.

Our findings confirm and expand on the gene expression of *FSHR* and *LHCGR* in cumulus cells during IVM [17], where *FSHR* was significantly downregulated, and *LHCGR* was significantly upregulated in parallel with oocyte maturation. Several TGF-β members, including AMH (gene and protein), inhibin-B, total inhibin, and GDF9, were significantly downregulated in the spent medium when the oocyte reached the MII stage compared to those remaining at the GV and, to a lesser extent, MI stages. This reduction in gene expression and synthesis likely reflects FSHR downregulation and a diminished FSH response.

Furthermore, *LHCGR* was positively associated with the resumption of meiosis and negatively associated with GDF9 secretion from the oocyte, supporting previous studies in rodents where exogenously added GDF9 prevented *LHCGR* expression and downregulated hCG-stimulated progesterone secretion in granulosa cells during culture [24, 25]. Thus, in vivo, one function of the secretion of GDF9 in high concentrations from immature oocytes in the SAFs is hypothesized to be preventing *LHCGR* expression and premature advancement of meiosis. Additionally, for the first time, we show that *LHCGR* expression on cumulus cells was positively associated with the concentration of inhibin-A in the spent medium while being negatively associated with inhibin-B concentration. This finding is notable as inhibin-A has been shown to enhance oocyte maturation in human and primate oocytes during IVM [26, 27], reinforcing its role in advancing oocyte maturation.

**Fig. 3:**
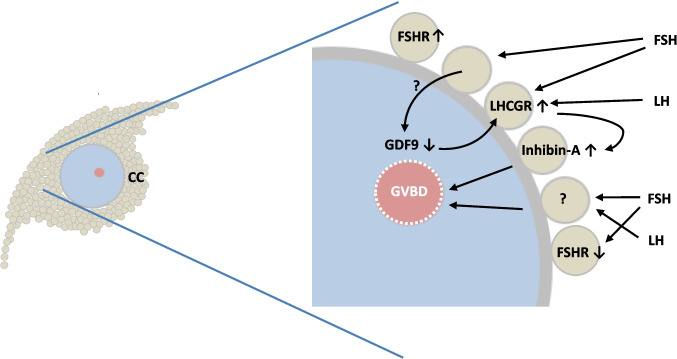
Working hypothesis for the regulation of oocyte human maturation in vitro of cumulus oocyte complexes from human small antral follicles: Relatively high FSHR expression is present on cumulus cells from human small antral follicles (28). High concentrations of FSH (> 75 IU/L) stimulate cumulus cells to cause immature oocytes to downregulate GDF9 secretion. Reduced GDF9 secretion allows cumulus cells to upregulate FSH-stimulated LHCGR expression. LH in the IVM medium (plus potentially FSH) causes augmented secretion of inhibin-A, which in turn stimulates germinal vesicle breakdown (GVBD) and oocyte maturation in combination with other yet unidentified positive signals secreted from cumulus cells by FSH and LH. FSHR expression becomes downregulated in COCs in which MII transition takes place probably caused by the high concentration of FSH in the medium, which, however, does not take place in COCs that remain in the GV stage. CC, cumulus cells; FSHR, FSH receptor; LHCGR, LH receptor; GDF9, growth and differentiation factor 9. Up and downregulation is depicted by arrows pointing up and down, respectively

Collectively, this suggests that key components of regulating human oocyte maturation involve releasing the brake by reducing the inhibitory effect of GDF9, enabling LHCGR expression, and accelerating the process by LH-induced secretion of inhibin-A, plus stimulation by other potential FSH-induced hormones (Fig. 3). This is a working hypothesis that requires further experimental data, including testing whether oocyte maturation can be improved by adding these hormones to the culture medium during IVM.

Under in vivo conditions, GDF9 secretion decreases tenfold in follicular fluid as follicles progress from SAFs to pre-ovulatory follicles over a 14-day period [28]. This time span is likely shortened during the IVM period due to the high expression of FSHR on cumulus cells from human SAFs and the high concentration of FSH in the IVM medium, collectively ensuring a downregulation of GDF9. This, in turn, allows LHCGR expression and may partly explain the accelerated oocyte maturation in vitro.

Importantly, FSH alone, without LH, also stimulated oocyte maturation to the same extent as the combination of the two gonadotropins. This likely reflects that FSH stimulates other hormones that advance oocyte maturation independently of LHCGR expression. Recently, we demonstrated that the IGF signaling pathway, including the gene expression of IGF2, was augmented in cumulus cells from COCs that sustain MII transition (unpublished). We were unable to measure components of the IGF signaling in the present study due to limited sample material (i.e., 25 µl per droplet), but this is an area where additional studies are warranted. Furthermore, the effects of FSH include *LHCGR* induction on cumulus cells, suggesting that additional beneficial effects from *LHCGR* stimulation are integral to the signal transduction pathways active during IVM, which is likely to include the secretion of other factors, such as amphiregulin [29, 30]. Collectively, it is hypothesized that a combined effect of FSH and LH advances both the nuclear and cytoplasmatic maturation and the overall viability and embryonic developmental capacity of these oocytes, but further studies are needed to corroborate this.

Overall, this study sets the stage for developing an IVM platform that creates a suitable hormonal environment to support oocyte maturation locally, with appropriate stimulation of cumulus cells. A more profound understanding of the mechanisms at play during human IVM will provide opportunities to optimize the viability and competence of oocytes beyond the remarkedly high 70–75% MII maturation rate as obtained in the present study using concentrations of FSH alone (100 IU/L) and FSH (100 IU/L) plus LH (100 IU/L) in L-COCs by further modifying the hormonal environment with exogenous hormones.

The current data does not provide information on the mechanisms involved in *FSHR* downregulation during IVM in cumulus cells supporting MII transition. However, it may be speculated that the high supraphysiological concentrations of FSH in the IVM medium result in a massive stimulation of the FSHR causing internalization and downregulation. Oocytes where the FSH stimulation results in LHCGR expression before or in connection with FSHR downregulation are likely to receive appropriate stimulation for oocyte maturation.

It appears that FSH maintains stimulation of cumulus cells when *FSHR* escapes downregulation (i.e., cumulus cells in which the oocyte remains in the GV stage), resulting in augmented concentrations of hormones like AMH, inhibin-B [31], and total inhibin in the spent medium. As cumulus cells from these follicles are less capable of stimulating oocyte maturation, it is likely that these substances have a minor direct effect on promoting oocyte maturation themselves. In contrast, *LHCGR* expression is associated with MII transition and positively correlated with inhibin-A, while GDF9 concentration is significantly downregulated (Fig. 2).

The number of somatic cells surrounding the oocyte during IVM (i.e., NO, S-COCs, or L-COCs) significantly augmented hormonal secretions like AMH, inhibin-B, and total inhibin in the spent medium where the oocyte remained in the GV stage, whereas levels were uniform in media from oocytes that sustained MII transition. This difference became more pronounced when the concentration of FSH in the medium was increased and LH added. These observations fit with sustained high *FSHR* expression in cumulus cells where the oocyte remains in the GV stage. For the oocyte-secreted GDF9, the picture is different, with relatively low levels in NO, peaking in S-COCs, and intermediate levels in spent medium from MII oocytes. This may reflect that the NO originates in follicles that have initiated atresia [32], while S-COCs may derive from less mature follicles, and L-COCs may represent follicles more prone to develop LHCGR expression.

Including FSH in the IVM medium (even at a concentration of 10 IU/L) downregulated GDF9 secretion to the spent medium where oocytes remained in the GV-stage without an apparent effect from LH, while cumulus cells from oocytes that resumed meiosis showed constant low levels of GDF9, irrespective of FSH concentration. This is probably an important event for IVM to become efficient and may reflect a pre-existing condition of the cumulus cells and/or an FSH-induced downregulation of GDF9 allowing for *LHCGR* expression. In contrast, AMH was produced in higher concentrations relative to the number of somatic cells and was overall higher in spent medium in which the oocyte remained in the GV-stage as compared to the MII group of oocytes, but in spent media from oocytes that reached the MII stage, concentrations were similar irrespective of media composition. This is likely a secondary effect of FSHR downregulation and most likely reflects that AMH has only a minor, if any, effect on regulating oocyte maturation. Like AMH, inhibin-B was downregulated in spent media in which the oocyte reached the MII stage and upregulated in parallel with the number of somatic cells, but the concentration was independent of the media composition, again suggesting that its synthesis is a secondary event to FSHR downregulation.

The validity of data for inhibin-A is hampered by the low number of valid observations, but the picture seems to be reverted as compared to AMH and inhibin-B as concentrations in spent media in which the oocyte reached the MII stage tended to increase compared to media from GV-stage oocytes. Furthermore, FSH and LH appear to stimulate the synthesis of inhibin-A in cumulus cells. This fits with the pattern that inhibin-A is secreted during the normal menstrual cycle, starting to become upregulated as follicles are selected at around 8–10 mm in diameter and begin to express LHCGR [31, 33]. Our observations are in line with a previous study in normal healthy women in which recombinant LH was found to stimulate inhibin-A but not inhibin-B in pre-ovulatory follicles, and it was suggested that LH is the primary stimulator of inhibin-A in preovulatory follicles [34]. In human follicular fluid collected during final maturation of follicles, inhibin-A increases during the first 17 h after a bolus injection of hCG concomitant with the occurrence of meiotic resumption [4]. In granulosa cells from these follicles, a significant upregulation of *INHBA* gene expression occurs within the first 12 h, while there is a downregulation of *INHBB* and *INHA* [4]. Collectively, this suggests that inhibin-A is associated with the progression of human oocyte maturation and may function differently from inhibin-B. Amphiregulin is also significantly upregulated during the final maturation of follicles in vivo, peaking at around 200 ng/mL in the follicular fluid [4]. At the same time point, inhibin-A concentrations reach around 400 ng/mL, which is one to two orders of magnitude higher than the concentrations measured in the spent medium in this study. This indicates that increasing concentrations of these and potentially other hormones could enhance both the quality and frequency of oocytes that reach the MII stage. However, this hypothesis requires further investigation to be substantiated.

In terms of clinical implementation of an IVM platform, it is preferable that the hormones are produced in situ by stimulating the cumulus cells instead of being added exogenously. This approach avoids the extensive safety testing and approval process required for exogenously produced proteins by regulatory authorities, while also providing a deeper understanding of the mechanisms involved. It is noteworthy that the AMH and GDF9 concentrations achieved in this study resemble those measured in follicular fluid from human SAFs [35, 36], suggesting that an intrafollicular-like milieu is created in the droplet used for IVM, potentially enhancing the developmental competence of the oocytes. Future IVM protocols could aim to enhance LHCGR expression in cumulus cells by modulating GDF9 production, for instance, through FSH stimulation. A stepwise approach—where COCs are first exposed to 100 IU/L FSH before LH stimulation—may optimize oocyte maturation. However, this strategy implies that immediate activation of LHCGR after its expression on cumulus cells (following FSH stimulation) may be less effective than delayed LH stimulation. This hypothesis warrants further evaluation in upcoming experiments.

One limitation of the present study is that secretion of steroids was not studied. The small droplets used for IVM are covered by a layer of mineral oil, which is very lipophilic. Studies with radiolabeled estradiol and progesterone in the medium showed that more than two thirds of the steroids diffused into the oil layer during a 24-h incubation period (unpublished data), which has been also previously shown in other species [37, 38]. Therefore, steroid measurements will be imprecise and were omitted.

Another limitation is the number of samples included in this study. As the IVM method is increasingly used clinically to enhance fertility preservation in connection with OTC, opportunities to conduct studies with a purely scientific purpose become reduced. Additionally, only L-COCs were used for gene expression analysis, which have demonstrated better maturation rates than S-COCs and NO (Table 1.). Thus, oocytes from other groups may exhibit distinct gene expression patterns and be regulated by different mechanisms during maturation. Due to limitations in sample material from the spent medium, we were unable to measure also the concentration of amphiregulin, which has shown to be an important hormone stimulating human oocyte maturation in vivo [4, 30], and in vitro [29], and the synthesis and importance of amphiregulin in this setting need further evaluation in new studies. Furthermore, how FSH signals from the IVM medium are transmitted to the oocyte via cumulus cells remains unclear. In vivo, transzonal projection (TZP) retraction in mouse cumulus cells can be triggered by FSH during follicle growth [39] or by EGFR ligands transmitting LHCGR signaling after the LH surge [40]. In vitro, studies in animal models show that TZPs retract spontaneously after COC removal, taking approximately 16 h in mice [40], though the timing in humans remains unknown.

We speculate that in COCs from SAFs, TZPs remain functional long enough to mediate FSH signaling, while their spontaneous loss may also contribute to meiotic resumption—possibly explaining why ~ 40% of oocytes reach MII without gonadotropins. Although we did not assess TZPs in this study, their role in human oocyte maturation in vitro warrants further investigation.

In conclusion, the present study demonstrates remarkably high maturation rates of L-COCs when appropriately stimulated with FSH and LH. Concurrent downregulation of *FSHR* gene expression and upregulation of *LHCGR* are closely associated with oocyte maturation. Downregulation of GDF9 secretion is linked to *LHCGR* upregulation and positively correlates with MII transition. The cumulus cells are induced to produce high concentrations of TGF-β members like AMH, inhibin-B, and inhibin-A, which aligned with the gene expression profiles of the cumulus cells. Collectively, the regulation of oocyte maturation from human SAFs may involve FSH-induced GDF9 downregulation, allowing *LHCGR* expression (“releasing the brake”), which is positively associated with inhibin-A secretion. This process advances oocyte maturation (“accelerated oocyte maturation”), probably in collaboration with other FSH-induced factors, such as amphiregulin and IGF2.

## Supplementary Information

Below is the link to the electronic supplementary material.

Supplementary file1 (DOCX 15 KB)

Supplementary file2 (DOCX 29 KB)

Supplementary file3 (DOCX 27 KB)

Supplementary file4 (DOCX 27 KB)

Supplementary file5 (DOCX 21 KB)

## Data Availability

Datasets generated during and/or analyzed during the current study are not publicly available but are available from the corresponding author on reasonable request.
