# Supplementary material for: Regulating human oocyte maturation in vitro: a hypothesis based on oocytes retrieved from small antral follicles during ovarian tissue cryopreservation
Source: J Assist Reprod Genet. 2025 Apr 22;42(5):1461–72. doi: 10.1007/s10815-025-03483-9 (PMC12167398; doi:10.1007/s10815-025-03483-9)
Supplement: Supplementary file 1 — Supplementary file1 (DOCX 15 KB) [file 10815_2025_3483_MOESM1_ESM.docx]

| **Substance** | **Cat. No.** | **Manufacturer** | **Dynamic Range** | **LoD** | **Precision % CV**  **(Measured Concentration)** |
| --- | --- | --- | --- | --- | --- |
| Inhibin-A | AL-123 | Anshlab, Houston, Tx, USA | 9.9 – 1188 pg/mL | 5.45 pg/mL | 6.2% (101.3 pg/mL), 5.5% (344.9 pg/mL) |
| Inhibin-B | AL-107 | Anshlab, Houston, Tx, USA | 12.7 – 1390 pg/mL | 1.6 pg/mL | 5.6% (99.4 pg/mL), 5.0% (308.1 pg/mL) |
| Total inhibin | AL-134 | Anshlab, Houston, Tx, USA | 8.3-525 pg/mL | 1.0 pg/mL | 4.5% (20.6 pg/mL), 3.9% (69.8 pg/mL) |
| GDF9 | AL-176 | Anshlab, Houston, Tx, USA | 48 – 5800 pg/mL | 3 pg/mL | 4.3% (392 pg/mL), 6.9% (3902 pg/mL) |
| AMH | AL-124 | Anshlab, Houston, Tx, USA | 7.6-1091 pg/mL | 1 pg/mL | 4.3% (64.1 pg/mL), 4.7% (186.4 pg/mL) |

**Supplementary table 1** Characteristics of ELISA kits used.

LOD: Limit of detection
