# Supplementary material for: Regulating human oocyte maturation in vitro: a hypothesis based on oocytes retrieved from small antral follicles during ovarian tissue cryopreservation
Source: J Assist Reprod Genet. 2025 Apr 22;42(5):1461–72. doi: 10.1007/s10815-025-03483-9 (PMC12167398; doi:10.1007/s10815-025-03483-9)
Supplement: Supplementary file 2 — Supplementary file2 (DOCX 29 KB) [file 10815_2025_3483_MOESM2_ESM.docx]

**Supplementary Table 2** Concentration of GDF9 (ng/ml) in spent medium after human IVM (mean ±SEM)

| **Cumulus size** | **GV** | **M1** | **M2** | **Total** | **P-value** |
| --- | --- | --- | --- | --- | --- |
| Naked oocytes | 0.3 ± 0.04^c1, A1^  n=6 | 2.8 ± 0.4^c1,c2^  n=4 | 0.8 ± 0.3^c2^  n=9 | 1.1 ± 0.3^C1^  n=19 | <0.001 |
| Small-COCs | 5.8 ± 1.7^A1, A2^  n=13 | 7.8 ± 2.4^a2^  n=7 | 2.2 ± 0.5^a2^  n=20 | 4.3 ± 0.8^C1,C2^  n=40 | <0.02 |
| Large-COCs | 2.5 ± 0.4^b1, A2^  n=20 | 3.8 ± 0.8^b2^  n=5 | 1.2 ± 0.2^b1,b2^  n=43 | 1.8 ± 0.2^C2^  n=68 | <0.001 |
| P-value | <0.01 | NS | <0.05 | <0.001 |  |
| **IVM treatment** |  |  |  |  |  |
| No GT | 7.1 ± 1.6^b1,B1 ,B2 ;B3^  n=12 | 5.3 ± 2.5  n=3 | 1.5 ± 0.4^b1,^  n=14 | 4.2 ± 0.9^A1,A2^  n=29 | <0.01 |
| FSH10 | 1.7 ± 0.5^c1,B1^  n=13 | 8.7 ±3.1^c1,c2^  n=5 | 1.1 ±0.2^c2^  n=21 | 2.3 ± 0.6  n=39 | <0.001 |
| FSH100 | 1.7 ± 0.4^B2^  n=8 | 2.9 ± 0.5  n=3 | 1.6 ±0.5  n=20 | 1.7 ± 0.3^A1^  n=31 | NS |
| FSH100 + LH100 | 1.2 ± 0.6^B3^  n=6 | 3.2 ± 0.7  n=5 | 1.6 ±0.5  n=17 | 1.8 ± 0.3^A2^  n=28 | NS |
| Total | 3.3 ± 0.7^b1^  n=39 | 5.3 ± 1.2^c1^  n=16 | 1.4 ± 0.2^b1,c1^  n=72 | 2.5 ± 0.3  n=127 | <0.001 |
| P-value | <0.001 | NS | NS | <0.05 |  |

Tukey post-hoc analysis. Uppercase letters designate comparisons within a column while lowercase letters designate comparisons within a row. Letters with the same number are compared. A and a: P<0.05; B and b: P<0.01; C and c: P<0.001; NS: not significant (P > 0.05). No GT: group with no gonadotropins; FSH10: group with 10 IU/L rFSH; FSH100: group with 100 IU/L rFSH; FSH100+LH100: group with both 100 IU/L rFHS and 100 IU/L rLH; GV: germinal vesicle; M1: metaphase I; M2: metaphase II.
