# Supplementary material for: Regulating human oocyte maturation in vitro: a hypothesis based on oocytes retrieved from small antral follicles during ovarian tissue cryopreservation
Source: J Assist Reprod Genet. 2025 Apr 22;42(5):1461–72. doi: 10.1007/s10815-025-03483-9 (PMC12167398; doi:10.1007/s10815-025-03483-9)
Supplement: Supplementary file 3 — Supplementary file3 (DOCX 27 KB) [file 10815_2025_3483_MOESM3_ESM.docx]

**Supplementary Table 3** Concentration of AMH (ng/ml) in spent medium after human IVM (mean ±SEM)

| **Cumulus size** | **GV** | **M1** | **M2** | **Total** | **P-value** |
| --- | --- | --- | --- | --- | --- |
| Naked oocytes | 7.6 ± 3.9  n=2 | n=0 | 2.9 ± 1.2^A1^  n=4 | 5.4 ± 1.2  n=6 | NS |
| Small-COCs | 22.7 ± 4.4^a1^  n=11 | 11.4 ± 3.7  n=7 | 10.8 ± 2.4^a1, C1^  n=16 | 14.8 ± 2.1^B1^  n=34 | 0.03 |
| Large-COCs | 465 ±159^b1^  n=18 | 289 ± 79  n=5 | 99 ± 13^b1,A1,C1^  n=43 | 213 ± 48^B1^  n=66 | 0.002 |
| P-value | NS | NS | P<0.05 | P<0.001 |  |
| **IVM treatment** |  |  |  |  |  |
| No GT | 152 ± 49  n=11 | 4.4 ± 1.4  n=2 | 73 ± 23  n=11 | 103 ± 26  n=24 | NS |
| FSH10 | 77 ± 30  n=8 | 173 ± 95  n=5 | 72 ± 27  n=15 | 91 ± 24  n=28 | NS |
| FSH100 | 350 ± 229^c1^  n=6 | 31 ± 22  n=3 | 44 ± 14^c1^  n=19 | 108 ± 53  n=28 | <0.05 |
| FSH100 + LH100 | 709 ± 435  n=6 | 188 ± 131  n=3 | 102 ± 20  n=17 | 252 ± 108  n=26 | NS (P=0.06) |
| Total | 279 ± 100^b1^  n=31 | 118 ± 48  n=13 | 72 ± 11^b1^  n=62 | 138 ± 31  n=106 | 0.013 |
| P-value | NS | NS | NS | NS |  |

Tukey post-hoc analysis. Uppercase letters designate comparison within a column while lowercase letters designate comparisons within a row. Letters with the same number are compared. A and a: p<0.05; B and b: p<0.01; C and c: p<0.00; NS: not significant (P > 0.05). No GT: group with no gonadotropins; FSH10: group with 10 IU/L rFSH; FSH100: group with 100 IU/L rFSH; FSH100+LH100: group with both 100 IU/L rFHS and 100 IU/L rLH; GV: germinal vesicle; M1: metaphase I; M2: metaphase II.
