# Supplementary material for: Regulating human oocyte maturation in vitro: a hypothesis based on oocytes retrieved from small antral follicles during ovarian tissue cryopreservation
Source: J Assist Reprod Genet. 2025 Apr 22;42(5):1461–72. doi: 10.1007/s10815-025-03483-9 (PMC12167398; doi:10.1007/s10815-025-03483-9)
Supplement: Supplementary file 4 — Supplementary file4 (DOCX 27 KB) [file 10815_2025_3483_MOESM4_ESM.docx]

**Supplementary Table 4** Concentration of Inhibin-B (ng/ml) in spent medium after human IVM (mean ±SEM)

| **Cumulus size** | **GV** | **M1** | **M2** | **Total** | **P-value** |
| --- | --- | --- | --- | --- | --- |
| Naked oocytes | BDL | N=0 | BDL | 0 |  |
| Small-COCs | 7.3 ± 1.8^B1^  n=9 | 2.7 ± 0.7^B1^  n=4 | 3.8 ± 0.9^B1^  n=8 | 5.4 ± 1.0^B1^  N=21 | NS |
| Large-COCs | 63 ± 11.9^b1,c1,B1^  n=19 | 30 ± 5.8^b1,B1^  n=9 | 12 ± 1.0^c1,B1^  n=56 | 25.5 ± 3.6^B1^  n=84 | <0.001 |
| P-value | <0.01 | <0.01 | <0.01 | <0.01 |  |
| **IVM treatment** |  |  |  |  |  |
| No GT | 32 ± 8.0  N=9 | n=0 | 17 ± 3.7  n=8 | 26 ± 5.0  n=17 | NS |
| FSH10 | 35 ± 14.1  n=7 | 25 ± 13.5  n=4 | 11 ± 2.2  n=15 | 20 ± 4.7  n=26 | NS (P = 0.08) |
| FSH100 | 44 ± 26.8  n=6 | 7± 2.7  n=3 | 11 ± 1.7  n=14 | 12 ± 3.1  n=23 | NS |
| FSH100 + LH100 | 82 ± 26.5^b1,c1^  n=6 | 26 ± 6.8^b1^  n=6 | 11 ± 1.4^c1^  n=27 | 20 ± 3.8  n=39 | <0.001 |
| Total | 46 ± 9.3^a1,c1^  n=28 | 23 ± 5.5^a1^  n=13 | 11 ± 1.0^c1^  n=64 | 21 ± 3.6  n=105 | <0.001 |
| P-value | NS | NS | NS | NS |  |

Tukey post-hoc analysis. Uppercase letters designate comparisons within a column while lowercase letters designate comparisons within a row. Letters with the same number are compared. A and a: P<0.05; B and b: P<0.01; C and c: P<0.001; NS: not significant (P > 0.05). BDL: below detection limit. No GT: group with no gonadotropins; FSH10: group with 10 IU/L rFSH; FSH100: group with 100 IU/L rFSH; FSH100+LH100: group with both 100 IU/L rFHS and 100 IU/L rLH; GV: germinal vesicle; M1: metaphase I; M2: metaphase II.
