# Supplementary material for: Regulating human oocyte maturation in vitro: a hypothesis based on oocytes retrieved from small antral follicles during ovarian tissue cryopreservation
Source: J Assist Reprod Genet. 2025 Apr 22;42(5):1461–72. doi: 10.1007/s10815-025-03483-9 (PMC12167398; doi:10.1007/s10815-025-03483-9)
Supplement: Supplementary file 5 — Supplementary file5 (DOCX 21 KB) [file 10815_2025_3483_MOESM5_ESM.docx]

**Supplementary Table 5** Concentration of Inhibins (ng/ml) in spent medium after IVM in relation to maturational status, oocyte type and media composition.

|  | **All Oocytes** | |  | **L-COCs** | |  | **All Oocytes** | | |  | **Media composition (IU/L)** | | | |
| --- | --- | --- | --- | --- | --- | --- | --- | --- | --- | --- | --- | --- | --- | --- |
|  | **GV** | **MII** |  | **GV** | **MII** |  | **NO** | **S-COC** | **L-COC** |  | **0 FSH** | **10 FSH** | **100 FSH** | **100 FSH + 100 LH** |
| **Inhibin-B** | 46 ± 9.3^c^  n=28 | 11 ± 0.9^c^  n=64 |  | 63 ± 11.9^c^  n=19 | 12 ± 1.0^c^  n=56 |  | 1.7 ± 0.03  n=2 | 5.4 ± 1.0^b^  n=19 | 25.5 ± 3.6^b^  n=84 |  | 26 ± 5.0  n=16 | 20 ± 4.7  n=26 | 12 ± 3.1  n=22 | 20 ± 3.8  n=36 |
| **Inhibin-A** | 3.8 ± 1.7  n=10 | 7.6 ± 1.0  n=33 (P=0.07) |  | 5.5 ± 2.2  n=7 | 8.3 ± 1.0  n=29 |  | 0.05  n=1 | 1.4 ± 0.5^b^  n=6 | 8.2 ± 0.9^b^  n=40 |  | 2.5 ± 0.8  N=3 | 5.8 ± 1.3  n=7 | 6.6 ± 1.3  n=12 | 9.9 ± 1.5  n=21  (P=0.09) |
| **Total-Inhibin** | 4.7 ± 1.3^a^  n=25 | 2.5 ± 0.3^a^  n=51 |  | 6.6 ± 1.7^b^  n=17 | 2.9 ± 0.3^b^  n=41 |  | 0.3 ± 0.04  n=2 | 0.7 ± 0.1^c^  n=19 | 4.2 ± 0.5^c^  n=63 |  | 2.2 ± 0.4  n=15 | 2.4 ± 0.5^a^ n=23 | 2.7 ± 0.8  n=23 | 5.4 ± 1.2^a^  n=23 |

Comparisons are made within each category of oocytes (i.e., all oocytes, large-COC, all oocytes and media composition) using either ANOVA or t-test. Within a row, same letters are compared. ^a^ P<0.05; ^b^ P<0.01; ^c^ P<0.001. GV: germinal vesicle; M1: metaphase I; M2: metaphase II; NO: naked oocytes, S-COCs: small cumulus-oocyte complexes (COCs); L-COCs: large COCs.
